# Interictal invasive very high-frequency oscillations in resting awake state and sleep

**DOI:** 10.1038/s41598-023-46024-z

**Published:** 2023-11-06

**Authors:** Karin Revajová, Vojtěch Trávníček, Pavel Jurák, Zuzana Vašíčková, Josef Halámek, Petr Klimeš, Jan Cimbálník, Milan Brázdil, Martin Pail

**Affiliations:** 1 Brno Epilepsy Center, Department of Neurology, member of ERN-EpiCARE, St Anne’s University Hospital and Medical Faculty of Masaryk University, Brno, 602 00 Czech Republic; 2 Central European Institute of Technology, Masaryk University, Brno, 602 00 Czech Republic; 3 Institute of Scientific Instruments, Czech Academy of Sciences, Brno, 602 00 Czech Republic; 4 International Clinical Research Center, St Anne’s University Hospital, Brno, 602 00 Czech Republic

**Keywords:** Neurological disorders, Epilepsy, Neuroscience, Neurology

## Abstract

Interictal very high-frequency oscillations (VHFOs, 500–2000 Hz) in a resting awake state seem to be, according to a precedent study of our team, a more specific predictor of a good outcome of the epilepsy surgery compared to traditional interictal high-frequency oscillations (HFOs, 80–500 Hz). In this study, we retested this hypothesis on a larger cohort of patients. In addition, we also collected patients' sleep data and hypothesized that the occurrence of VHFOs in sleep will be greater than in resting state. We recorded interictal invasive electroencephalographic (iEEG) oscillations in 104 patients with drug-resistant epilepsy in a resting state and in 35 patients during sleep. 21 patients in the rest study and 11 patients in the sleep study met the inclusion criteria (interictal HFOs and VHFOs present in iEEG recordings, a surgical intervention and a postoperative follow-up of at least 1 year) for further evaluation of iEEG data. In the rest study, patients with good postoperative outcomes had significantly higher ratio of resected contacts with VHFOs compared to HFOs. In sleep, VHFOs were more abundant than in rest and the percentage of resected contacts in patients with good and poor outcomes did not considerably differ in any type of oscillations. In conclusion, (1) our results confirm, in a larger patient cohort, our previous work about VHFOs being a specific predictor of the area which needs to be resected; and (2) that more frequent sleep VHFOs do not further improve the results.

## Introduction

In the surgical treatment of epilepsy refractory to medication, accurate localization and delineation of epileptogenic tissue in the brain is crucial. This precise determination of epileptogenic tissue remains, despite a remarkable progress in epilepsy research since its beginnings in 1905^1^, a significant challenge for epilepsy surgery programs nowadays. Standard surgery protocols based on, among other neuro-imaging techniques, invasive electroencephalography (iEEG) evaluations of the seizure-onset zone (SOZ, defined with intracranial ictal EEG activity of frequencies typically < 40 Hz), do not reach desirable postoperative effectiveness, as a considerable percentage (50%) of patients do not remain seizure-free after the surgery^2^. Generally, the goal is to find the epileptogenic zone, which is defined as the minimal amount of tissue necessary to remove in a patient to achieve seizure freedom^3^. Since this is a rather theoretical concept, which cannot be validated, we will be talking about biomarkers prognosticating surgical outcomes^4^.

In order to ameliorate the precision of the epilepsy surgery outcomes prognosis, scientific research has focused on high-frequency oscillations of frequencies 80–500 Hz (HFOs), which are believed to be precise biomarkers connected with epileptogenicity^5–9^. However, a recent study by Zweiphenning et al.^10^ has not shown a non-inferiority of HFO-guided neurosurgery to spike-guided epilepsy surgery.

Over the past more than a decade, high-frequency EEG oscillations of frequencies > 500 Hz (very high-frequency oscillations, VHFOs) have emerged as a promising, even more specific biomarker prognosticating outcomes in epilepsy surgery than HFOs^11^, which was also reported in a previous work of our team^12^.

The current study aimed to test our hypothesis that interictal VHFOs are a more precise biomarker prognosticating surgery outcome than HFOs on an augmented cohort of subjects with epilepsy refractory to medication (40 previously published subjects by Brázdil et al., 2017^12^ and 64 new patients to from a total of 104 subjects), using invasive interictal rest EEG recordings. However, as the occurrence of interictal VHFOs in rest iEEG recordings seems to be relatively low^12^, we also studied invasive sleep EEG recordings hypothesising that in these recordings, the presence of interictal VHFOs might be higher than in rest iEEG recordings.

## Methods

We adopted the same analytical approach and performed an identical presurgical evaluation of patients as in our previously published paper^12^. In our present study, we have developed two hypotheses regarding rest and sleep iEEG recordings (see above). Therefore, we divide this section into two parts, describing patients and methods in the rest and sleep analyses, respectively.

### Rest awake analysis

We analysed the data of 104 patients with drug-resistant epilepsy^13^—53 with focal temporal lobe epilepsy, 44 with extratemporal lobe epilepsy, one patient with both temporal and extratemporal, and 6 with unknown epilepsy region. There were fifty female and 54 male subjects. All patients provided an informed consent to participate in the study and underwent intracranial stereo-EEG (SEEG) monitoring. The study was approved by the ethics committees of Masaryk University and St. Anne’s University Hospital. All research was performed in accordance with the Declaration of Helsinki.

In each subject, we recorded a 30-min EEG in a wakeful resting state with intracerebral multi-contact platinum SEEG electrodes, each patient receiving 5 to 16 SEEG electrodes. The location and number of electrodes were selected based on clinical reasoning. Either DIXI or ALCIS electrodes were used. The technical parameters of the electrodes were as follows: a diameter of 0.8 mm; a contact length of 2 mm; an intercontact distance of 1.5 mm; a contact surface area of 5 mm^2^; a number of contacts being 5, 8, 10, 12, 15 and 18. All the electrodes were MRI compatible, and their position in the brain was verified by MRI or a combination of MRI and CT examination.

A battery-powered EEG acquisition system of 192 channels was used for the recordings, with a sampling rate of 5 kHz, dynamic range of ± 25 mV with 10 nV (24 bits). The recording reference used was the average of all intracranial signals. Standard epilepsy protocol units were used, with no special shield.

SignalPlant^14^ and custom python scripts were used for data processing.

21 subjects (11 females and 10 males)*,* met the criteria for further rest iEEG data analysis (ie, statistical analysis of postoperative outcome correlated with the resected contacts containing the studied events). The criteria were: interictal ripples (Rs, 80–200 Hz), fast ripples (FRs, 200–500 Hz), very-fast ripples (VFRs, 500–1000 Hz), and ultra-fast ripples (UFRs, > 1000 Hz) present in iEEG recordings (in other terms, HFOs and VHFOs both present in iEEG recordings of patients; ripples and fast ripples by their frequency range belong to HFOs; very-fast ripples and ultra-fast ripples according to their frequency range fall into the category of VHFOs), a surgical intervention (either a resection of epileptogenic tissue or thermocoagulation of the tissue, or both) and postoperative follow-up of at least 1 year. All these subjects underwent a complex presurgical evaluation, consisting of: scalp and invasive video-EEG monitoring, MR, PET, optional ictal SPECT imaging, medical history and neurological and neuropsychological examination. The subjects' characteristics are presented in Table 1*.* Subjects No. 003-040 belong to the cohort already published by Brázdil et al.^12^.

**Table 1:** Patient characteristics.

Subject no.	Gender	Age at seizure onset [yr]	Age at SEEG [yr]	Seizure type/No. per month	MRI signs	Type/Side of epilepsy	SOZ	Surgery/histopathology	Outcome,Engel stage [yr]
Patient characteristics—Rest analysis									
003	F	19	57	FIAS/2 + , FBTCS/6	Postischemic Lesions within L T-O and H	T/L	L H ant.	AMTR/Gliosis, Hemosiderin within T pole	IA (8)
007	F	16	33	FIAS/5	Bilateral HS	T/ bilat	R Amygdala, R and L H ant., R H post.	R AMTR/Negative	IIIA (3)
013	F	17	26	FIAS/12	Normal	T/L	L Gyrus Temporalis Medius	L AMTR/FCD Ib	IA (3)
014	F	28	56	FIAS/8	R HS	T/R	R Amygdala/H ant. and post.	R AMTR/Negative	IIIA (5)
015	M	1	40	FIAS/2 +	Hypotrophic L H	T/L	L H ant. and post.	L AMTR/Negative	IIA (9)
021	M	33	41	FIAS/30	Focal Hyperintensity R basal T	T/R	R H ant. and post., Lesion (FCD)	R AMTR/FCD IIIb, Ganglioglioma	IA (8)
031	M	31	37	FIAS/4	Normal	T/L	L H ant. and post., R H ant.	L AMTR/Negative	IB (8)
032	F	9	27	FIAS/5	L HS	T/L	L H ant. and post.	L AMTR/FCD IIIa	IIIA (1)
033	M	2	51	FIAS/3 +	R H Atrophy, slight changes of density	T/R	R H ant. and post.	R AMTR/HS	IA (8)
040	F	9	36	FIAS/5	Bilateral H Atrophy and Malrotation	T/R	R Gyrus Temporalis Superior, R H ant. and post.	R AMTR/Negative	IB (7)
043	F	13	17	FIAS/30 +	Normal	T/R	R H ant.	R AMTR/ HS type I	IA (2)
045	F	15	56	FIAS/3	Posttraumatic Lesions within L O	T/R	R H ant.	R AMTR/ HS type I	IA (2)
047	F	6	26	FIAS/15 +	R HS	T/R	R H ant.	R AMTR/ HS type I	IA (2)
083	F	25	32	FAS/45	FCD mesial R, upper Frontal Gyrus	E/R	R Gyrus Frontalis Medialis	Cortectomy/FCD IIa	IA (2)
084	M	32	35	FIAS/8	Cystic Lesions mesial Frontal	E/R	Lesion, R Nucleus Caudatus	Thermocoagulation	IB (2)
087	M	15	28	FIAS/6	Normal	T bilat	R and L Gyrus Parahip-pocampalis and H ant.	T R AMTR/ HS type I	IIIA (1)
092	M	5	41	FIAS/15 +	Normal	E/L	L Insula Posterior,L Gyri Temporales Transversi	Cortectomy/FCD IIa	IIIA (2)
Patient characteristics—Rest and sleep analysis									
088	M	14	28	FIAS/12 +	Normal	E/L	L Gyrus Lingualis	Thermocoagulation, Cortectomy T-O L	IB (2)
090	M	12	32	FIAS/2	Normal	E/R	R Gyrus Frontalis Medialis L H ant. and post.	Cortectomy/FCD IIa	IIIA (2)
100	F	8	33	FIAS/5	FCD L Supramarginal Gyrus	E/L	L Gyrus Supramarginalis	Cortectomy/FCD Ia	IIIA (2)
102	M	3	51	FIAS/2	R H Atrophy	T/R	R H ant.	R AMTR/ HS type I	IA (1,5)
Patient characteristics—Sleep analysis									
060	F	9	32	FIAS/3	Postoperative Changes L T	T/L	L Gyrus Fusiformis, L Gyrus Temporalis Inferior	Cortectomy/FCD IIb	IA (3)
066	M	12	28	FIAS/5 +	FCD dorsal part R Gyrus Temporalis Superior	T/R	R Gyrus Temporalis Medius	Cortectomy/FCD IIB, Nodular heterotophy	IVA (2)
067	F	1,5	21	FIAS/4 +	FCD R central area, Nodular Heterotopia R lateral ventricle	E/R	Lesion(FCD)	Cortectomy/FCD IIa	IVB (2)
071	F	5	25	FIAS/20 +	F L Post-encephalitic changes, L HS	T/L	L H ant. and post.	AMTR L/ HS type I	IA (2)
077	M	18	29	FIAS/100	Normal	E/L	L Insula post.	Thermocoagulation	IA (3)
081	M	0	45	FIAS/120 +	Hyperintense changes P-O bilat	E/R	R Cuneus	Cortectomy/FCD IIa	IIIA (2)
093	F	21	52	FIAS/5 +	R H Atrophy	E/R	R Gyri Orbitales, R Gyrus Frontalis Inferior	Thermocoagulation	IA (2)

AMTR, Anteromedial temporal resection; ant., Anterior; E, Extratemporal; F, Female; FAS, Focal aware seizure; FBTCS, Focal to bilateral tonic–clonic seizures; FCD, Focal cortical dysplasia; FIAS, Focal impaired awareness seizure; H, Hippocampus; HS, Hippocampal sclerosis; L, Left; M, Male; MRI, Magnetic resonance imaging; O, Occipital; + , Sporadic FBTCS; post., Posterior; R, Right; SEEG, Stereo-electroencephalography; SOZ, Seizure onset zone; T, Temporal; [yr], Years.

In terms of previous neurosurgical interventions, one patient had a vagal nerve stimulation system implanted before the surgery, with a poor clinical outcome. After the surgery, all 21 subjects were evaluated and divided into 2 categories—subjects with good and unfavourable (poor) clinical outcome, based on the Engel classification^15^ of the postoperative clinical seizure outcome. The group with good clinical outcome was comprised of subjects who met Class I criteria of the Engel classification. All other subjects (ie, Class II–IV) were categorised as having an unfavourable clinical outcomes. 13 patients met the criteria for a good outcome, out of whom 9 subjects were rated as seizure free (Engel IA) and 4 patients presented with auras only since surgery (Engel IB). 8 patients did not have a favourable outcome—7 patients were rated Engel IIIA (worthwhile seizures reduction), 1 patient was almost seizure-free (Engel II).

### Sleep analysis

We monitored 35 patients during a whole night of sleep. Of these subjects, 13 were females, and 22 were males, age distribution ranged between 21 and 58.

Average duration of the iEEG recording was 391 min (approx. 6,5 h) with 595 min being the longest and 192 min being the shortest time of the iEEG recorded.

We used the same method, ie, electrodes, EEG acquisition system, and software as for the rest study (see “Rest analysis”). Regarding the scoring of the sleep, additional three scalp electrodes covering the frontal, central, and parietal regions, electrodes for electrooculography and electromyography of the chin were used according to the 2015 criteria of the American Association of Sleep Medicine^16^. Sleep was scored manually in 30 s epochs in the scalp EEG by a sleep expert.

Eleven patients (5 females, 6 males) met the criteria for postoperative outcome correlation with resected contacts (identical to the criteria listed in rest analysis) in the sleep iEEG data. When dividing patients according to their postoperative outcomes, 6 patients fell into the good outcome group (5 in Engel IA category, 1in Engel IB), and 5 into the poor outcome group (3 patients in Engel IIIA category, one patient in IVA, and one patient in IVB Engel category). The characteristics of these patients are stated in Table 1.

As visible in the Table 1, the overlap of patients meeting the inclusion criteria for both rest and sleep analysis was small (4 patients), mainly due to the criterion of a necessity of UFRs presence in the iEEG recordings of patients. For the purpose of creating a more significant cohort of patients for a within-patient comparison of rest and sleep analysis, we created one additional cohort of patients, where the selection criteria were modified to only R, FRs, and VFRs present in both rest and sleep iEEG recordings (we omitted UFRs). This cohort contained twelve patients, 6 with positive and 6 with negative outcomes. All selection criteria and their impact on number of patients included in the statistical analysis are presented in the Figure S1 in the supplement to this manuscript.

### Data pre-processing, statistical analysis

**Figure 1:**
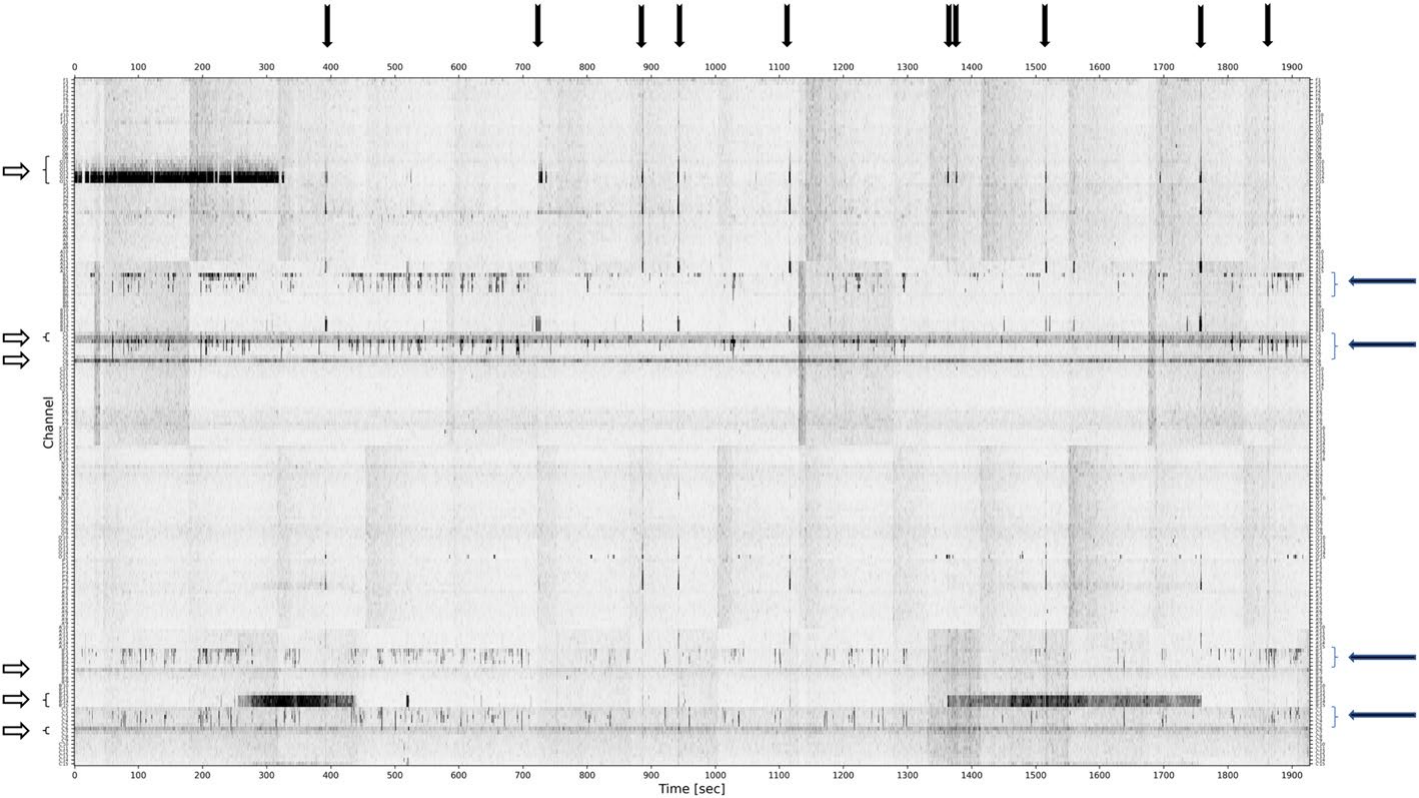
Power distribution matrix, rest iEEG. Very fast ripples (VFRs), freqency range (500–1000 Hz). Black arrow, Artifacts type 1—artificial signals spread through multiple electrodes. White arrow, Artifacts type 2—noise in the selected contact(s) during the whole recording. Blue arrow—VFR activity.

**Figure 2:**
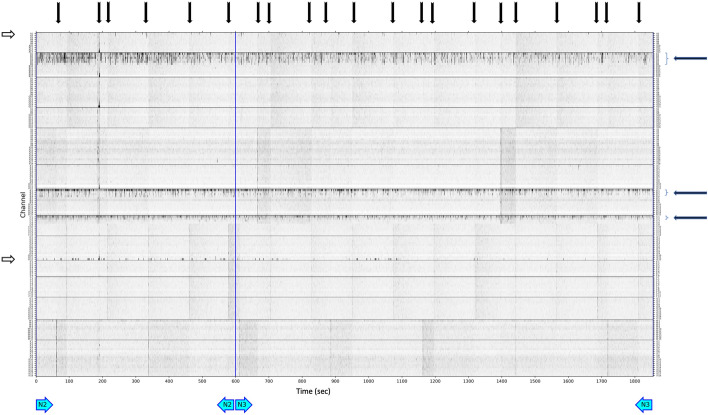
Power distribution matrix, sleep iEEG. Very fast ripples (VFRs), freqency range (500–1000 Hz). Black arrow, Artifacts type 1—artificial signals spread through multiple electrodes. White arrow, artifacts type 2—noise in the selected contact(s) during the whole recording. Blue arrow—VFR activity. N2—non-REM sleep stage 2; N3—non-REM sleep stage 3.

**Figure 3:**
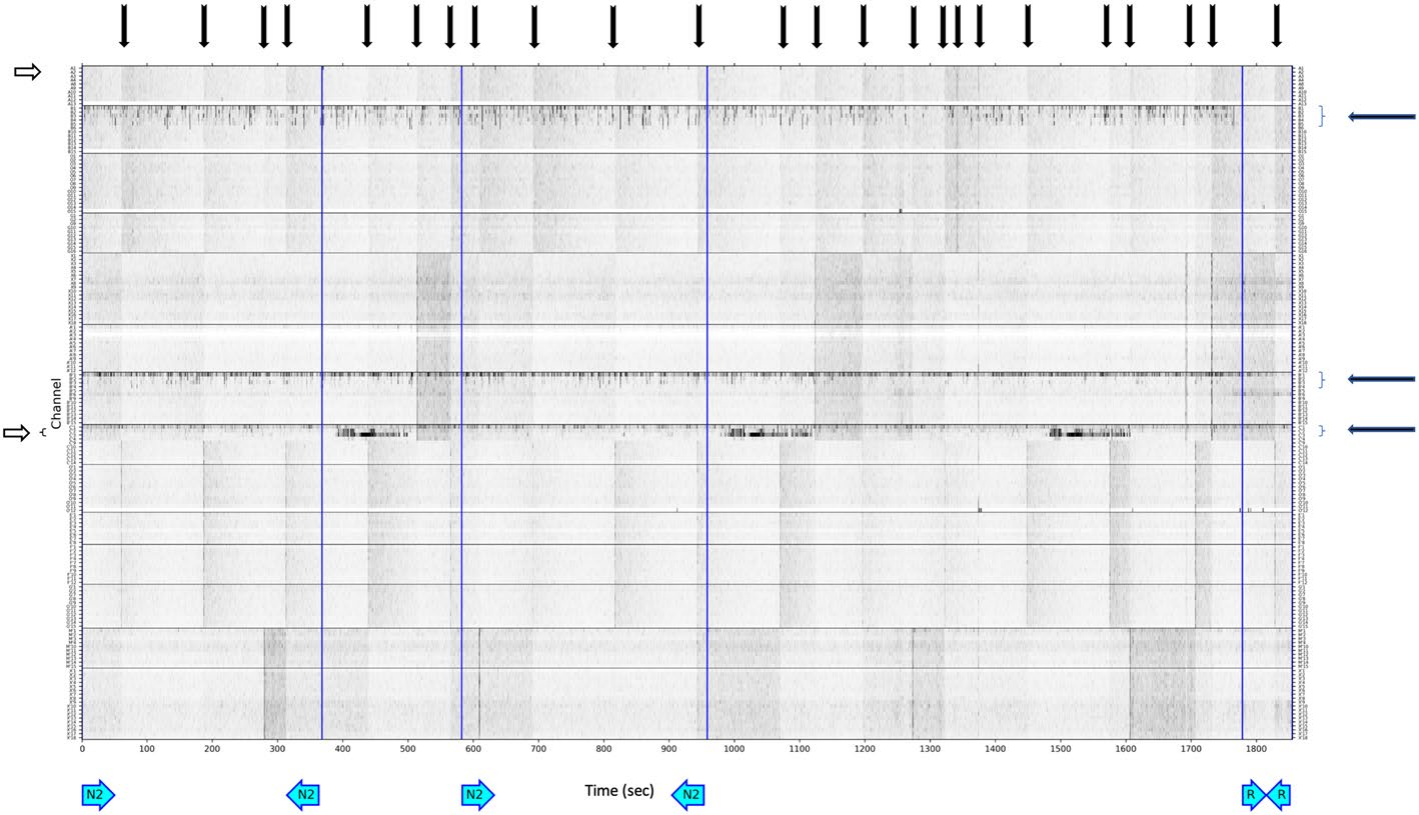
Power distribution matrix, sleep iEEG. Very fast ripples (VFRs), freqency range (500–1000 Hz). Black arrow, Artifacts type 1—artificial signals spread through multiple electrodes. White arrow, Artifacts type 2—noise in the selected contact(s) during the whole recording. Blue arrow—VFR activity. N2—non-REM sleep stage 2; R—REM sleep stage.

We performed an identical preprocessing of our data and statistical analysis in both sleep and rest iEEG data as in the previous study^12^. Original 5 kHz data were processed in 4 different frequency bands: Rs, FRs, VFRs, and UFRs. The power envelope was computed for each frequency band, and subsampled to 1 kHz. All recordings were referenced to the average of intracranial signals. Power envelopes were subsequently visualized as 2-dimensional power distribution matrices (PDM; where rows correspond to contacts and columns to a time, 30 min duration) for manual inspection. Figures 1, 2 and 3 show PDMs in rest and sleep in patient No. 071 (sleep) and No. 090 (rest). The goal of the manual inspection was to classify each channel either as with presence of HFOs/VHFOs or without the presence of HFOs/VHFOs. For the channel to be classified as with the presence of HFOs/VHFOs, it had to fulfill these principals:

- Dark thin (1 pixel) stripes present in the channel row
- Stripes are irregularly present during whole width of PDM
- Stripes visible throughout all channels (vertical lines) are artifacts
- If not sure whether stripe represents oscillation or artifact, inspect the original signal.

The visual inspection of PDMs and identification of channels with HFOs and VHFOs was conducted by two independent reviewers independently without any previous information about the type of resection, postoperative outcome, or resected contacts. Cohen's Kappa was calculated to report interrater variability. After processing all PDMs, the reviewers investigated mismatched channels together and came to an agreement (Cohen’s Kappa evaluated beforehand).

Resected and non-resected contacts were determined by the fusion of MRI and CT with implanted electrodes and post-surgical MRI, performed 3 months after the resection. Contacts were considered resected if found within the surgical cavity on the post-surgical MRI.

Correlations between the different types of oscillations and the post-surgical outcome were calculated as a percentage of removed contacts (see the Eq. (2) below) and the ratio between the number of removed and non-removed contacts (Eq. (1)) separately for each type of oscillations and also for SOZ (in SOZ, the contacts with the first ictal change were used in calculations, as identified by two independent epileptologists).

1 $$\mathrm{Ratio}(\mathrm{ev})=\frac{\mathrm{No}.\mathrm{rem}\left(\mathrm{ev}\right)-\mathrm{No}.\mathrm{Nrem}\left(\mathrm{ev}\right)}{\mathrm{No}.\mathrm{rem}\left(\mathrm{ev}\right)+\mathrm{ No}.\mathrm{Nrem}\left(\mathrm{ev}\right)}$$

2 $$\mathrm{Percentage}(\mathrm{ev})=100\mathrm{ x}\frac{\mathrm{No}.\mathrm{rem}\left(\mathrm{ev}\right)}{\mathrm{No}.\mathrm{rem}\left(\mathrm{ev}\right)+\mathrm{ No}.\mathrm{Nrem}\left(\mathrm{ev}\right)}$$

ev = type of oscillations (either R, FR, VFR, or UFR); No.rem = number of removed contacts with (ev); No.Nrem(ev) = nonremoved contacts with (ev)

Numerical data are given as median [interquartile range]. Wilcoxon rank-sum test was used to test differences between groups with good and bad outcome, Wilcoxon sign-rank test for within patient comparison of VFRs in rest and sleep, threshold for statistical significance was set to 0.05.

Number of subjects in rest analysis was 21 and in sleep analysis 11. Parameters have non-normal distribution given by quantization (number of contacts) and by low number of subjects.

## Results

### Rest awake iEEG analysis

49 patients had interictal VFRs present in their iEEG recordings, and in 36 subjects ultra-fast ripples were detected (data available for UFR analysis were 92 in subjects). 21 subjects (13 with good postsurgical outcome) that met our criteria (see “patients and methods”) were included in further statistical analysis of postoperative outcome in relation to the resection of contacts with different types of oscillations (Rs, FRs, UFRs, VFRs).

Interictal very high-frequency phenomena possessed the same characteristics as in our first study by Brázdil et al.^12^, regarding their spatial distribution and rate—97% of contacts where UFRs were present had also VFRs, 84% of contacts with UFRs had FRs and 83% occurred with Rs. 91% VFR contacts in rest occurred with FR and 85% occurred with ripples. The average number of contacts with Rs was 20 [29], FRs 12 [14], VFRs 6 [3], and UFRs 3 [1] per patient. The interrater variability was 0.917 for VFR and 0.791 for UFR. Thus, they occurred mostly in regions with Rs and FRs, and the reduction in the number of contacts occurred with increasingly higher frequencies of oscillations.

The localization of rest VHFOs in relation to brain structures was, contrary to our first findings^12^, not limited to mesial-temporal structures—we did record oscillations of very high frequencies in other regions of the brain, as in Usui et al., 2010^11^, such as in parietal, temporal-occipital and frontal cortex. VFRs in extra-mesial-temporal structures were found in 8 patients (out of 49) and UFRs in 5 patients (out of 36). The structures where VHFOs (VFRs and/or UFRs) were found were: gyrus frontalis medialis and frontal operculum, gyrus temporalis superior (in 2 patients), gyrus temporalis medialis, temporal operculum, praecuneus and gyrus supramarginalis. If not stated otherwise, each of these extra-mesial-temporal VHFOs were found only in 1 patient and we have not found any patient with more than one region with extra-mesial-temporal VHFOs.

66.7% of contacts with VFRs and 100% of UFRs were removed in patients with good outcome, whereas in the group with unfavourable outcome, 26.7% and 16.7% of contacts respectively were removed, respectively, the difference between good and poor outcomes reached statistical significance. The results of the statistical analysis of our data are visualised in Table 2*.*

**Table 2:** Correlation between resected contacts and postoperative outcome—rest analysis.

Outcome	SOZ	R	FR	VFR	UFR
Percentage (contacts) resected Median [IQR]					
Good	100.0 [0]	27.66 [30.48]	57.14 [44.23]	66.67 [69.23]	100.0 [42.86]
Poor	40.0 [13.98]	17.33 [14.40]	20.0 [21.58]	26.79 [41.66]	16.67 [50.0]
* p*-value	0.0001	0.13	0.056	0.034	0.005
Ratio (contacts) resected Median [IQR]					
Good	1.0 [0]	− 0.45 [0.61]	0.14 [0.88]	0.33 [1.38]	1.0 [0.86]
Poor	− 0.20 [0.28]	− 0.66 [0.30]	− 0.60 [0.43]	− 0.46 [0.84]	− 0.67 [1.0]
* p*-value	0.0001	0.13	0.057	0.034	0.005

FR, Fast ripple; IQR, Inter-quartile range; R, Ripple; SOZ, Seizure-onset zone; UFR, Ultra fast ripple; VFR, Very fast ripple.

In ripples and fast ripples, 27.7% and 57.1% respectively, of contacts were removed in patients with good outcome, with no statistical significance between the good and poor outcome groups. Two patients with good postoperative outcome had no contacts with VHFOs resected (No. 084 and 088).

### Sleep iEEG analysis

In the sleep iEEG analysis, there were 35 subjects included in the study, however, data available for analysis were only in 27 subjects. We did not detect VFRs in 4 patients and UFRs in 10 patients. As such, VFRs were present in 85% of sleep iEEG recordings and UFRs were detected in almost 63%.

Eleven patients (6 with good postoperative outcome) met our criteria for further statistical analysis. More clinical information about these subjects are provided in Table 1. These subjects are not identical to those included in the rest analysis, as not all patients in the rest analysis group underwent the sleep iEEG monitoring, in some subjects, the rest data are not of sufficient quality and/or unavailable, and, in several subjects (No. 060, 067, 077), VHFOs were not detected during the rest but were detected in sleep.

The average number of contacts with sleep Rs was 60 [11.5] per patient, 25 [17.5] with FRs, 6 [8.5] with VFRs and 3 [5.5] with UFRs, the interrater variability was 0.914 for VFRs and 0.843 for UFRs. Concerning the match of UFRs and R, FRs and VFRs, 92% of contacts with UFRs had also VFRs, 69% of contacts with UFRs had FRs and 61% had Rs. As for VFRs, 73% of contacts with VFRs had also FRs and 68% had Rs.

Regarding the percentage of removed contacts in relation to postoperative outcome (see Table 3), in the good outcome group, 100% of the contacts in SOZ were removed. In Rs, FRs, VFRs, and UFRs it was 11.9%, 19.2%, 7.1%, and 8.3%, respectively. In addition to that, ratio and percentage of resected contacts in poor outcome subjects compared to good outcome subjects almost did not differ in R (9.6%) and FR (16.1%). The results of the comparison of good and poor outcome groups' resection did not reach statistical significance in any type of oscillation, not even in SOZ.

**Table 3:** Correlation between resected contacts and postoperative outcome—sleep analysis.

Outcome	SOZ	R	FR	VFR	UFR
Percentage (contacts) resected Median [IQR]					
Good	100 [33.33]	11.89 [13.37]	19.16 [27.04]	7.14 [26.65]	8.33 [19.7]
Poor	60 [76.92]	9.62 [18.18]	16.13 [17.11]	0.0 [17.95]	0.0 [0]
* p*-value	0.477	0.831	0.790	0.740	0.396
Ratio (contacts) resected Median [IQR]					
Good	1 [0.67]	− 0.76 [0.27]	− 0.62 [0.54]	− 0.86 [0.53]	− 0.83 [0.38]
Poor	0,2 [1.54]	− 0.81 [0.36]	− 0.68 [0.34]	− 1 [0.36]	− 1.0 [0]
* p*-value	0.477	0.820	0.781	0.740	0.396

FR, Fast ripple; IQR, Inter-quartile range; R, Ripple; SOZ, Seizure-onset zone; UFR, Ultra fast ripple; VFR, Very fast ripple.

The localization of VHFOs (VFRs and UFRs) in the investigated subjects corresponded mainly to the mesial-temporal structures of the brain, but in three patients, VHF phenomena were also present in other brain structures corresponding to the eloquent cortex—gyri of the temporo-occipital, occipital and frontal and medial-frontal cortex.

We also visually studied the presence of VHFOs in the individual sleep stages—they were mostly localized in non-REM (N) stages (in 100% of inspected iEEG recordings), being especially abundant in N2 stage, but they were also considerably present in N1. In the REM phase of sleep, they were present to a remarkably lower extent than in the non-REM phases (in 30% of inspected iEEG recordings) and they were difficult to notice.

Number of patients with a good quality both rest and sleep iEEG recordings for a within-patient analysis was 26. Out of these patients, 14 patients (53.85%) had rest VFRs in their rest iEEG recordings, whereas sleep VFRs were present in 21 (80.77%) sleep iEEG recordings.

Number of contacts with rest VFRs was 123, in sleep it was 129. According to Wilcoxon sign-rank test, there was no statistical significance between the amount of contacts in rest and sleep (*p* = 0.85). Concerning the match of the contacts with rest and sleep VFRs, 62 contacts were found only in rest, 68 contacts only in sleep and 61 contacts in rest and in sleep.

The investigated cohort of patients with both rest and sleep VFRs (without the patients having only rest or only sleep VFRs) consisted of 12 patients, 6 patients with positive outcomes and 6 patients with negative outcomes. Patients with good outcomes had 46.4% of contacts with rest VFRs resected and 15.4% of contacts with sleep VFRs. The difference between percentages of removed contacts with VFRs in these patients was not statistically significant (*p* = 0.068), however, it is close to the significance level of 0.05 and shows a trend in the data (see Table 4).

**Table 4:** Correlation between resected contacts and postoperative outcome—VFRs rest and sleep analysis.

Outcome	Rest	Sleep	*p*-value_2_
Percentage (contacts) resected Median [IQR]			
Good	46.43 [22.98]	15.38[41.03]	0.068
Poor	0 [27.27]	0[13.64]	–
* p*-value_1_	0.15	0.52	–
Ratio (contacts) resected Median [IQR]			
Good	0.07[0.25]	− 0.69[0.82]	0.068
Poor	− 1[0.55]	− 1[0.27]	–
* p*-value_1_	0.15	0.52	–

IQR, Inter-quartile range; *p*-value_1_, Significance of the differences between percentages of removed contacts with VFRs in good and poor outcome patients for rest and sleep respectively; *p*-value_2_, Significance of the differences between percentages of removed contacts with rest and sleep VFRs in good outcome patients; VFRs, Very fast ripples.

## Discussion

In this study, we had two goals: the first was to retest the hypothesis of interictal very high-frequency EEG oscillations being a more specific biomarker prognosticating surgical outcome than the traditionally used HFOs, on a cohort of 104 patients. In the analysis of the rest iEEG recordings, we proved VHFOs to be a more specific prognostic biomarker of epilepsy surgery than HFOs. The classification of HFOs is normally based on identifying channels with abnormally higher rates but the identification of the threshold is individual to every patient. VHFOs present a different approach. These phenomena are more specific in prognosticating surgical outcome but less sensitive, meaning that if they are present in a certain brain area and this area is resected, they are very precise in prognosticating good surgery outcomes. Therefore, they do not replace HFOs, but rather complement them.

The numbers of patients with rest UFRs in iEEG recordings were limited, however, the absence of channels with UFRs is a poor prognostic factor that may reflect suboptimal spatial sampling of the epileptogenic zone or multifocality, rather than an inherently low sensitivity of UFRs.

The second goal was to advance our research by studying VHFOs’ presence in sleep iEEG recordings hypothesising that in sleep, a higher number of VHFOs might be present than in rest recordings.

The occurrence of VFRs and UFRs in sleep iEEG recordings in our study was more prominent than during rest—in the within-patient analysis of rest and sleep VFRs, 80.8% of patients had VFRs in their sleep iEEG recordings and 53.9% of them had VFRs in their rest iEEG recordings (regarding UFRs, they were present in 39% in the rest and in 63% in the sleep iEEG recordings). The difference between the number of contacts with rest and sleep VFRs was not significant (123 contacts with rest vs. 129 contacts with sleep VFRs), however, the match of rest versus sleep contacts with VFRs was only in 61 contacts. According to these results, sleep VHFOs are present in a higher number of patients, but the number of channels with sleep and rest VFRs did not significantly differ and the match between rest and sleep contacts with VFRs was only in about half of contacts.

**Figure 4:**
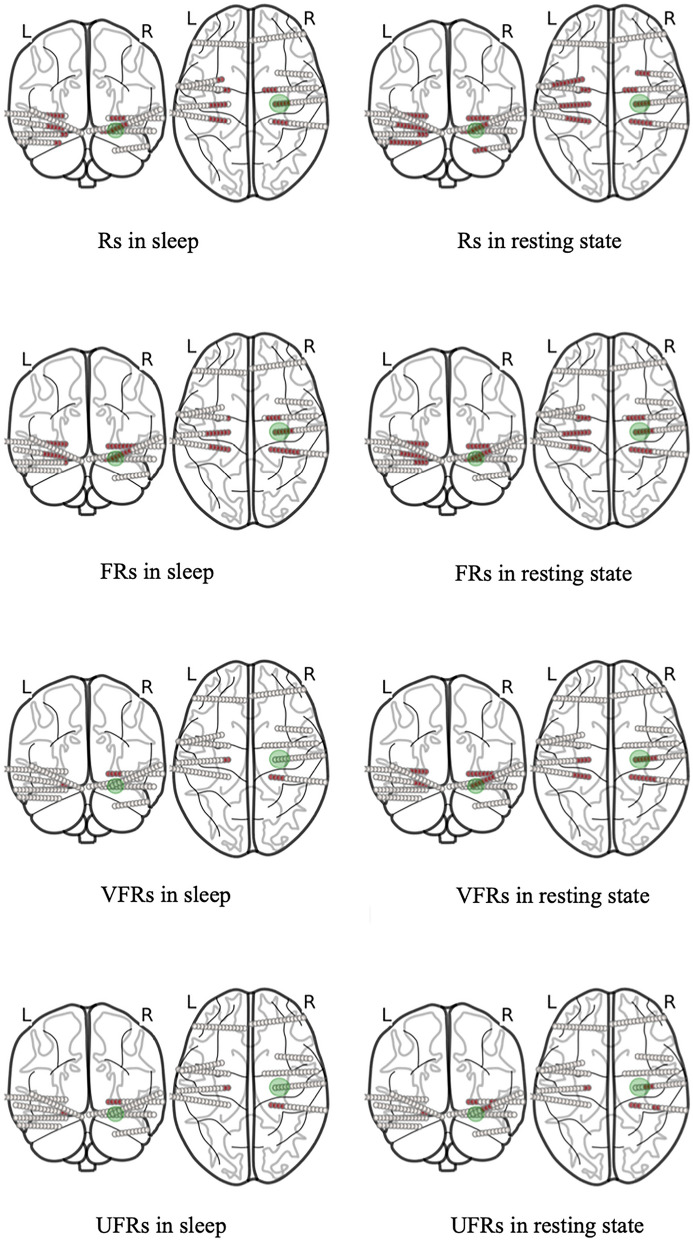
Visualisation of the results of rest and sleep study on patient n. 102. Green zone = SOZ; Red points = contacts on electrodes with either Rs, FRs, VFRs or UFRs; The reduction of the number of contacts with high-frequency phenomena from Rs to UFRs in both rest and sleep study is shown; Rest VHFOs better match with SOZ than rest HFOs; Rest VHFOs correspond better to SOZ than sleep VHFOs.

There was no proven statistical significance between the resected contacts in good and poor outcome groups, neither in HFOs nor in VHFOs. The analysed dataset also did not reach statistical significance between good and poor outcome groups in SOZ resection, which was probably due to the small number of studied subjects (11), as well as complexity of epilepsy cases included in the sleep study. This result does not change the impact of the conclusions from the sleep study, as the validity of results does not depend on the SOZ precise definition of the epileptogenic zone. In the sleep study, we were interested in the possibility of definition of the epileptogenic tissue by sleep VHFOs, but our aim was not to compare the precision of localisation of this tissue of SOZ versus VHFOs. We can however observe a trend of 100% of contacts' resection in SOZ in good outcomes, whereas only in 8–19% in HFOs and VHFOs, which also suggests SOZ's better precision in definition of the epileptogenic tissue even in this cohort of patients. In addition to that, the results of the within-patient analysis of rest and sleep VFRs also correspond with the trends from the rest and sleep analysis groups—the percentage of removed contacts in within-patient analysis in patients with good outcomes was higher in rest VFRs (46.4%) compared to sleep VFRs (15.4%). To sum up, VHFOs are occurring more frequently in sleep than in rest, as we hypothesised, but sleep VHFOs as such are not specific enough phenomena for the definition of epileptogenic tissue. See the visualisation of our results in the Fig. 4*.*

As the presence of very high-frequency oscillations in the sleep iEEG recordings was relatively frequent, but the channels positive with VHFOs in sleep did not fully correspond to those in rest (61 contacts with sleep VFRs matched with rest VFRs; total number of 123 contacts with rest VFRs), it is probable that what we have recorded were epileptogenic VHFOs as well as VHF oscillations corresponding to normal brain function. This theory could also be supported by the finding of VHFOs in the mesial-temporal (i.e. usually epileptogenic) structures as well as in some parts of the brain corresponding to the eloquent cortex. However, in line with our previous work, the detection of VHFOs in the archicortical areas (notably the hippocampus) clearly dominated and VHFOs are more frequent in patients suffering from temporal lobe epilepsies^12^.

We hereby refer to one of the most crucial problems in high-frequency oscillations (frequencies 80 Hz–2 kHz) analysis in general—a possible recording of both physiological/non-epileptogenic and pathological VHF phenomena during sleep and the need to differentiate between those two phenomena to achieve the successful delineation of epileptogenic tissue. The overlap of presumed physiological and pathological HFOs (80–500 Hz) has already been described by some authors^17–20^. The presence of physiological HFOs during sleep has been studied by Nonoda et al.^21^ in sensorimotor-visual cortex and by Buzsáki and Silva^19^ in hippocampi during memory consolidation in slow wave sleep and in neocortex via thalamic stimulation during non-REM sleep stage 2. The occurrence of VHFOs in primary motor and sensory areas in relation to the stimulation of the median nerve was reported by Sakura et al.^22^ followed by Cao et al.^23^, stimulating both the median and ulnar nerves.

From the pathogenetic point of view, it is assumed that pathological HFOs are generated by the simultaneous firing of small cell groups interconnected on a network level^24,25^.

Liu et al.^26^ and Cimbalnik et al.^27^ studied two types of HFOs—physiological and epileptogenic. The distinction between these two types of HFOs in both studies was performed automatically, by unsupervised machine learning techniques, based not only on the rate and frequency of EEG oscillations but on repetitive waveform morphology of epileptogenic HFOs (Liu’s work—the eloquent cortex was prone to produce waveforms of a rather random morphology, contrary to the epileptogenic tissue which tended to produce similar waveform morphology) and on multiple iEEG features (Cimbalnik—working with HFO features, univariate and bivariate features). Von Ellenrieder et al.^28^ proposed other methods by studying the presence of physiological and pathological HFOs in different sleep stages, and Nonoda et al.^21^, working with HFOs coupling with slow waves in sleep.

### Study limitation

In our study, the definition of the presence of R, FR, VFR, and UFR in iEEG recordings is based on the visual analysis of PD matrices, which did not include quantitative thresholding to distinguish between contacts with the presence and non-presence of HF or VHF events. However, even with the use of visual evaluation, the presence of these events on some contacts were interpreted considering the context—only VHFO rates outstanding from the surrounding noise level were included in the analysis. This visual analysis remains a relatively demanding, time-consuming process, depending on (the level of experience of) the reviewer and hence, prone to errors. However, PD matrices provide a straightforward way of displaying the distribution of oscillations at different frequencies in an approximately 30-min segment of iEEG recording at 5 kHz. This procedure corresponds to a standard clinical practice for identifying SOZ using iEEG, with a manual inspection of mainly preictal recordings. The essential reason for using PD matrices is that a validated algorithm for an automated identification VHFOs is not yet available. Such automated detectors have already been developed in the field of HFOs (80–500 Hz), as the automated analysis of EEG signals of frequencies in the range of 80–500 Hz proposed by Chaibi et al.^29^, or software such as MEEGIPS-A^30^, or CS algorithm^31^. Also, as iEEG recordings in sleep were longer than in rest, it might introduce a limitation in comparison between the number of contacts with HFOs and VHFOs in resting awake state and sleep.

Visual reviewing of PD matrices in search of VHFOs may not guarantee complete independence from the subjective evaluation, but we must also consider that even the process of defining SOZ is based on a subjective visual inspection of hours of video EEG recordings to locate the epileptogenic tissue. In addition to that, we should also regard the process of localization of the SOZ itself, which is the definition of this zone with ictal EEG—a procedure which consists of, in some cases, gradual withholding of the patients’ antiseizure medication, waiting until the patient presents with a seizure (days to weeks, or it might never happen), as well as awaiting a specific type of seizure. Identification of interictal very high-frequency phenomena are much less time-consuming (30 min of rest EEG recording needed) and patient-burdening (no need to wait for the seizures) than the process of standard identification of SOZ.

To conclude, VHFOs can be found in a limited number of patients, however, the study clearly showed that the presence of very high-frequency oscillations during wakeful rest provides a more specific value as a biomarker prognosticating surgical outcome than the presence of HFOs. Yet, VHFOs do not replace HFOs, but rather complement them. The occurrence of VHFOs in sleep is higher than in rest, however, the distinction of probably non-pathological VHFOs will be necessary in order to reach satisfactory specificity when prognosticating outcome of epilepsy surgery with sleep VHFO analysis.

### Supplementary Information

Supplementary Figure S1.

## Data Availability

Data available on request from the authors (Karin Revajová, Vojtěch Trávníček).
