# Supplementary figures and images for: Interictal invasive very high-frequency oscillations in resting awake state and sleep
Source: Sci Rep. 2023 Nov 6;13:19225. doi: 10.1038/s41598-023-46024-z (PMC10628183; doi:10.1038/s41598-023-46024-z)

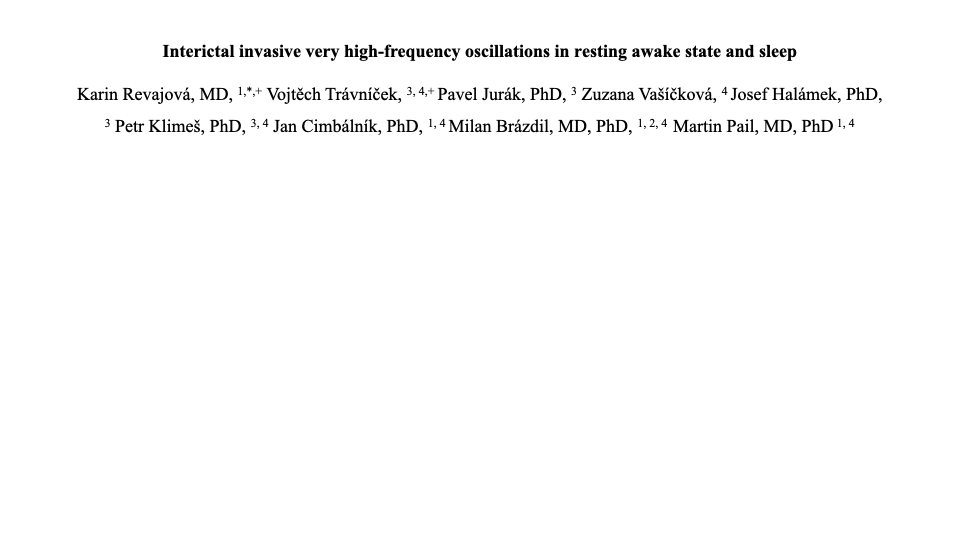

Supplement: Supplementary file 1 — Supplementary Figure S1. [file 41598_2023_46024_MOESM1_ESM.zip › Figure S1.tiff]
